# 3-(4-Chloro­phen­yl)quinazolin-4(3*H*)-one

**DOI:** 10.1107/S1600536811030935

**Published:** 2011-08-11

**Authors:** M. Gnana Ruba Priya, T. Srinivasan, K. Girija, N. Ravi Chandran, D. Velmurugan

**Affiliations:** a Department of Chemistry, CNK Reddy College of Pharmacy, Sastra University, Thanjavur 613 402, India; b Centre of Advanced Study in Crystallography and Biophysics, University of Madras, Guindy Campus, Chennai 600 025, India; c Mother Theresa Postgraduate & Health Science, Puducherry 605 006, India; d Sastra University, Thanjavur 613 402, India

## Abstract

In the title compound, C_14_H_9_ClN_2_O, the quinazoline unit is essentially planar, with a mean deviation from the least-squares plane defined by the ten constituent ring atoms of 0.027 (2) Å. The dihedral angle between the mean plane of the quinazoline ring system and the 4-chloro­phenyl ring is 44.63 (5)°. In the crystal, mol­ecules are linked by inter­molecular C—H⋯N and C—H⋯O hydrogen bonds, forming infinite chains of alternating *R_2_^2^*(6) dimers and *R_2_^2^*(14) ring motifs.

## Related literature

For the synthesis of the title compound, see: Priya *et al.* (2011 ▶). For related structures, see: Li & Feng (2009 ▶); Li *et al.* (2010 ▶). For the biological activity of quinazoline derivatives, see: Wolfe *et al.* (1990 ▶); Tereshima *et al.* (1995 ▶); Pandeya *et al.* (1999 ▶). For graph-set notation see: Bernstein *et al.* (1995 ▶).
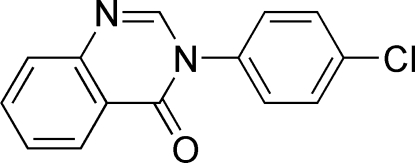


## Experimental

### 

#### Crystal data

C_14_H_9_ClN_2_O
*M* *_r_* = 256.68
Monoclinic, 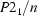

*a* = 16.9531 (8) Å
*b* = 3.9290 (3) Å
*c* = 17.2740 (8) Å
β = 91.626 (3)°
*V* = 1150.14 (12) Å^3^
*Z* = 4
Mo *K*α radiation
μ = 0.32 mm^−1^
*T* = 293 K
0.24 × 0.22 × 0.20 mm

#### Data collection

Bruker SMART APEXII area-detector diffractometer
Absorption correction: multi-scan (*SADABS*; Bruker, 2008 ▶) *T*_min_ = 0.926, *T*_max_ = 0.938
11055 measured reflections
2920 independent reflections
1870 reflections with *I* > 2σ(*I*)
*R*_int_ = 0.042

#### Refinement

*R*[*F* ^2^ > 2σ(*F* ^2^)] = 0.043
*wR*(*F* ^2^) = 0.133
*S* = 1.01
2920 reflections
163 parameters
H-atom parameters constrained
Δρ_max_ = 0.27 e Å^−3^
Δρ_min_ = −0.22 e Å^−3^

### 

Data collection: *APEX2* (Bruker, 2008 ▶); cell refinement: *SAINT* (Bruker, 2008 ▶); data reduction: *SAINT*; program(s) used to solve structure: *SHELXS97* (Sheldrick, 2008 ▶); program(s) used to refine structure: *SHELXL97* (Sheldrick, 2008 ▶); molecular graphics: *ORTEP-3* (Farrugia, 1997 ▶); software used to prepare material for publication: *SHELXL97* and *PLATON* (Spek, 2009 ▶).

## Supplementary Material

Crystal structure: contains datablock(s) global, I. DOI: 10.1107/S1600536811030935/lx2195sup1.cif

Structure factors: contains datablock(s) I. DOI: 10.1107/S1600536811030935/lx2195Isup2.hkl

Supplementary material file. DOI: 10.1107/S1600536811030935/lx2195Isup3.cml

Additional supplementary materials:  crystallographic information; 3D view; checkCIF report

## Figures and Tables

**Table 1:** Hydrogen-bond geometry (Å, °)

*D*—H⋯*A*	*D*—H	H⋯*A*	*D*⋯*A*	*D*—H⋯*A*
C7—H7⋯N1^i^	0.93	2.47	3.281 (2)	145
C13—H13⋯O1^ii^	0.93	2.37	3.145 (2)	140

Symmetry codes: (i) 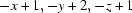; (ii) 
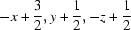
.

**Fig. 1.**
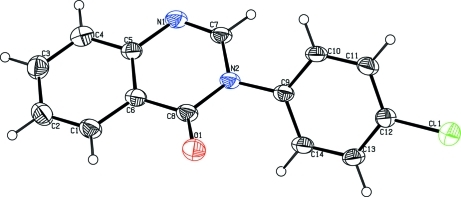
The molecular structure of the title compound, showing displacement ellipsoids drawn at the 30% probability level. H atoms are presented as a small spheres of arbitrary radius.

**Fig. 2.**
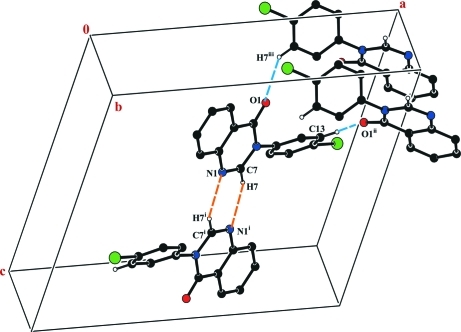
A view of the C—H···N and C—H···O hydrogen bonds (dotted lines) in the crystal structure of the title compound. [Symmetry codes: (i) - x + 1, - y + 2, - z + 1; (ii) - x - 3/2, y + 1/2, - z + 1/2; (iii) - x + 3/2, y - 1/2, - z + 1/2.]

## supplementary crystallographic information

### Comment

4(3*H*)-Quinazolinones are an important class of fused heterocycles with a wide range of biological activities such as anti–cancer (Wolfe *et al.*,1990), anti-inflammatory (Tereshima *et al.*,1995),  anti–HIV (Pandeya *et al.*, 1999). In addition to that, the quinazolinones exibit anti–bacterial and anti–bacterial and anti-fungal activities (Priya *et al.*, 2011).

In title molecule (Fig. 1), the quinazoline unit is essentially planar, with a mean deviation of 0.027 (2) Å from the least–squares plane defined by the ten constituent atoms. The dihedral angle formed by the 4-chlorophenyl ring and the mean plane of the quinazoline fragment is 44.63 (5)°. In the crystal packing (Fig. 2), molecules are linked by intermolecular C—H···N and C—H···O hydrogen bonds (Table 1). These hydrogen bonds are forming infinite chains of alternating *R_2_^2^*(6) dimer and *R_2_^2^*(14) ring motifs (Bernstein *et al.*, 1995) as shown in Fig. 2.

### Experimental

To an ice–cold solution of POCl_3_ in DMF was added anthranilic acid (0.01458 mole) and stirred for 5–10 min until TLC indicated the disappearance of anthranilic acid. The reaction-mixture was treated with the respective primary aromatic amine (0.01458 mol) and supported on anhydrous sodium sulfate (five times the weight of anthranilic acid) and exposed to microwave (BPL company) irradiation (600 W) for 2–4 min with 30 sec pulse. The reaction-mixture was quenched with water (50 ml) and extracted with ethyl acetate (2x50 ml). The organic layer was dried over anhydrous sodium sulfate, concentrated and purified by silica gel column chromatography (60–20 mesh) using hexane/EtOAc (7.5:2.5) as eluent to yield the pure product. Single crystals suitable for X–ray diffraction were prepared by slow evaporation of a solution of the title compound in methanol at room temperature.

### Refinement

Hydrogen atoms were placed in calculated positions with C—H = 0.93 Å and refined in riding model with fixed isotropic displacement parameter: *U*_iso_(H) = 1.2 *U*_eq_(C).

### Crystal data

**Table:**

C_14_H_9_ClN_2_O	*F*(000) = 528
*M**_r_* = 256.68	*D*_x_ = 1.482 Mg m^−^^3^
Monoclinic, *P*2_1_/*n*	Mo *K*α radiation, λ = 0.71073 Å
Hall symbol: -P 2yn	Cell parameters from 1025 reflections
*a* = 16.9531 (8) Å	θ = 1.7–28.5°
*b* = 3.9290 (3) Å	µ = 0.32 mm^−^^1^
*c* = 17.2740 (8) Å	*T* = 293 K
β = 91.626 (3)°	Block, colourless
*V* = 1150.14 (12) Å^3^	0.24 × 0.22 × 0.20 mm
*Z* = 4	

### Data collection

**Table:**

Bruker SMART APEXII area-detector diffractometer	2920 independent reflections
Radiation source: fine-focus sealed tube	1870 reflections with *I* > 2σ(*I*)
graphite	*R*_int_ = 0.042
ω and φ scans	θ_max_ = 28.5°, θ_min_ = 1.7°
Absorption correction: multi-scan (*SADABS*; Bruker, 2008)	*h* = −22→21
*T*_min_ = 0.926, *T*_max_ = 0.938	*k* = −5→5
11055 measured reflections	*l* = −23→23

### Refinement

**Table:**

Refinement on *F*^2^	Primary atom site location: structure-invariant direct methods
Least-squares matrix: full	Secondary atom site location: difference Fourier map
*R*[*F*^2^ > 2σ(*F*^2^)] = 0.043	Hydrogen site location: difference Fourier map
*wR*(*F*^2^) = 0.133	H-atom parameters constrained
*S* = 1.01	*w* = 1/[σ^2^(*F*_o_^2^) + (0.0705*P*)^2^ + 0.0848*P*] where *P* = (*F*_o_^2^ + 2*F*_c_^2^)/3
2920 reflections	(Δ/σ)_max_ = 0.001
163 parameters	Δρ_max_ = 0.27 e Å^−^^3^
0 restraints	Δρ_min_ = −0.22 e Å^−^^3^

### Special details

**Table:**

Geometry. All esds (except the esd in the dihedral angle between two l.s. planes) are estimated using the full covariance matrix. The cell esds are taken into account individually in the estimation of esds in distances, angles and torsion angles; correlations between esds in cell parameters are only used when they are defined by crystal symmetry. An approximate (isotropic) treatment of cell esds is used for estimating esds involving l.s. planes.
Refinement. Refinement of F^2^ against ALL reflections. The weighted R-factor wR and goodness of fit S are based on F^2^, conventional R-factors R are based on F, with F set to zero for negative F^2^. The threshold expression of F^2^ > 2sigma(F^2^) is used only for calculating R-factors(gt) etc. and is not relevant to the choice of reflections for refinement. R-factors based on F^2^ are statistically about twice as large as those based on F, and R- factors based on ALL data will be even larger.

### Fractional atomic coordinates and isotropic or equivalent isotropic displacement parameters (Å^2^)

**Table:**

	*x*	*y*	*z*	*U*_iso_*/*U*_eq_	
Cl1	0.91232 (3)	0.26102 (17)	0.49055 (3)	0.0650 (2)	
O1	0.61097 (8)	0.3729 (5)	0.25686 (8)	0.0644 (5)	
C1	0.46221 (11)	0.6243 (6)	0.19774 (10)	0.0517 (5)	
H1	0.4933	0.5149	0.1619	0.062*	
C2	0.38879 (13)	0.7399 (6)	0.17572 (11)	0.0578 (5)	
H2	0.3704	0.7118	0.1249	0.069*	
C3	0.34179 (12)	0.8996 (6)	0.22962 (12)	0.0575 (5)	
H3	0.2916	0.9752	0.2148	0.069*	
C4	0.36884 (11)	0.9464 (6)	0.30462 (11)	0.0525 (5)	
H4	0.3370	1.0538	0.3402	0.063*	
C5	0.44358 (10)	0.8339 (5)	0.32744 (9)	0.0419 (4)	
C6	0.49087 (10)	0.6695 (5)	0.27370 (9)	0.0426 (4)	
C7	0.54095 (11)	0.7942 (5)	0.42122 (9)	0.0445 (4)	
H7	0.5590	0.8353	0.4717	0.053*	
C8	0.56863 (10)	0.5452 (5)	0.29697 (9)	0.0448 (4)	
C9	0.67020 (10)	0.5459 (5)	0.40206 (8)	0.0405 (4)	
C10	0.68006 (10)	0.3971 (5)	0.47428 (9)	0.0455 (4)	
H10	0.6363	0.3521	0.5039	0.055*	
C11	0.75439 (11)	0.3154 (5)	0.50233 (10)	0.0486 (5)	
H11	0.7613	0.2197	0.5513	0.058*	
C12	0.81859 (10)	0.3767 (5)	0.45721 (10)	0.0452 (4)	
C13	0.80960 (10)	0.5292 (6)	0.38568 (9)	0.0480 (5)	
H13	0.8535	0.5725	0.3562	0.058*	
C14	0.73563 (10)	0.6173 (5)	0.35806 (9)	0.0452 (4)	
H14	0.7293	0.7240	0.3102	0.054*	
N1	0.47068 (8)	0.8923 (5)	0.40344 (8)	0.0477 (4)	
N2	0.59244 (8)	0.6340 (4)	0.37290 (7)	0.0407 (4)	

### Atomic displacement parameters (Å^2^)

**Table:**

	*U*^11^	*U*^22^	*U*^33^	*U*^12^	*U*^13^	*U*^23^
Cl1	0.0495 (3)	0.0838 (5)	0.0615 (3)	0.0035 (3)	−0.0001 (2)	0.0062 (3)
O1	0.0570 (8)	0.0892 (13)	0.0473 (7)	0.0129 (8)	0.0066 (6)	−0.0291 (7)
C1	0.0586 (11)	0.0583 (14)	0.0383 (9)	−0.0053 (10)	0.0062 (8)	−0.0070 (8)
C2	0.0656 (12)	0.0634 (15)	0.0440 (10)	−0.0081 (10)	−0.0057 (9)	0.0006 (9)
C3	0.0517 (11)	0.0605 (15)	0.0602 (11)	−0.0006 (10)	−0.0027 (9)	0.0063 (10)
C4	0.0504 (10)	0.0526 (14)	0.0550 (10)	−0.0007 (9)	0.0110 (8)	−0.0012 (9)
C5	0.0440 (9)	0.0444 (12)	0.0377 (8)	−0.0070 (7)	0.0090 (7)	−0.0022 (7)
C6	0.0484 (9)	0.0436 (12)	0.0362 (8)	−0.0077 (8)	0.0083 (7)	−0.0036 (7)
C7	0.0466 (9)	0.0546 (13)	0.0329 (8)	−0.0053 (8)	0.0117 (7)	−0.0093 (7)
C8	0.0485 (10)	0.0515 (13)	0.0349 (8)	−0.0036 (8)	0.0095 (7)	−0.0097 (8)
C9	0.0447 (9)	0.0437 (12)	0.0334 (8)	−0.0052 (8)	0.0085 (6)	−0.0051 (7)
C10	0.0477 (10)	0.0545 (13)	0.0349 (8)	−0.0097 (8)	0.0131 (7)	0.0009 (8)
C11	0.0542 (11)	0.0560 (14)	0.0359 (8)	−0.0071 (9)	0.0062 (7)	0.0047 (8)
C12	0.0441 (9)	0.0493 (12)	0.0424 (9)	−0.0026 (8)	0.0050 (7)	−0.0003 (8)
C13	0.0453 (10)	0.0566 (14)	0.0428 (9)	−0.0072 (9)	0.0138 (7)	0.0022 (8)
C14	0.0513 (10)	0.0506 (12)	0.0343 (8)	−0.0054 (8)	0.0109 (7)	0.0021 (8)
N1	0.0475 (8)	0.0582 (11)	0.0378 (7)	0.0000 (7)	0.0116 (6)	−0.0109 (7)
N2	0.0419 (7)	0.0492 (10)	0.0315 (6)	−0.0021 (6)	0.0088 (5)	−0.0074 (6)

### Geometric parameters (Å, °)

**Table:**

Cl1—C12	1.7353 (18)	C7—N2	1.377 (2)
O1—C8	1.217 (2)	C7—H7	0.9300
C1—C2	1.369 (3)	C8—N2	1.405 (2)
C1—C6	1.397 (2)	C9—C10	1.384 (2)
C1—H1	0.9300	C9—C14	1.391 (2)
C2—C3	1.392 (3)	C9—N2	1.440 (2)
C2—H2	0.9300	C10—C11	1.375 (3)
C3—C4	1.374 (3)	C10—H10	0.9300
C3—H3	0.9300	C11—C12	1.378 (2)
C4—C5	1.389 (3)	C11—H11	0.9300
C4—H4	0.9300	C12—C13	1.378 (3)
C5—N1	1.398 (2)	C13—C14	1.373 (3)
C5—C6	1.401 (2)	C13—H13	0.9300
C6—C8	1.452 (3)	C14—H14	0.9300
C7—N1	1.281 (2)		
			
C2—C1—C6	120.52 (18)	N2—C8—C6	114.17 (14)
C2—C1—H1	119.7	C10—C9—C14	120.00 (16)
C6—C1—H1	119.7	C10—C9—N2	120.19 (14)
C1—C2—C3	119.81 (18)	C14—C9—N2	119.79 (15)
C1—C2—H2	120.1	C11—C10—C9	120.16 (15)
C3—C2—H2	120.1	C11—C10—H10	119.9
C4—C3—C2	120.59 (19)	C9—C10—H10	119.9
C4—C3—H3	119.7	C10—C11—C12	119.38 (16)
C2—C3—H3	119.7	C10—C11—H11	120.3
C3—C4—C5	120.15 (18)	C12—C11—H11	120.3
C3—C4—H4	119.9	C13—C12—C11	120.94 (16)
C5—C4—H4	119.9	C13—C12—Cl1	119.24 (13)
C4—C5—N1	119.17 (16)	C11—C12—Cl1	119.81 (14)
C4—C5—C6	119.56 (16)	C14—C13—C12	119.86 (16)
N1—C5—C6	121.26 (16)	C14—C13—H13	120.1
C1—C6—C5	119.36 (17)	C12—C13—H13	120.1
C1—C6—C8	120.40 (16)	C13—C14—C9	119.60 (16)
C5—C6—C8	120.24 (15)	C13—C14—H14	120.2
N1—C7—N2	126.39 (15)	C9—C14—H14	120.2
N1—C7—H7	116.8	C7—N1—C5	117.04 (14)
N2—C7—H7	116.8	C7—N2—C8	120.60 (14)
O1—C8—N2	120.72 (16)	C7—N2—C9	119.18 (13)
O1—C8—C6	125.10 (15)	C8—N2—C9	120.18 (13)
			
C6—C1—C2—C3	−0.9 (3)	C10—C11—C12—Cl1	−177.74 (16)
C1—C2—C3—C4	0.9 (3)	C11—C12—C13—C14	−1.0 (3)
C2—C3—C4—C5	−0.1 (3)	Cl1—C12—C13—C14	178.99 (16)
C3—C4—C5—N1	178.32 (19)	C12—C13—C14—C9	−1.1 (3)
C3—C4—C5—C6	−0.6 (3)	C10—C9—C14—C13	2.1 (3)
C2—C1—C6—C5	0.2 (3)	N2—C9—C14—C13	−179.22 (18)
C2—C1—C6—C8	179.71 (19)	N2—C7—N1—C5	−0.6 (3)
C4—C5—C6—C1	0.6 (3)	C4—C5—N1—C7	−177.73 (19)
N1—C5—C6—C1	−178.32 (18)	C6—C5—N1—C7	1.2 (3)
C4—C5—C6—C8	−178.96 (18)	N1—C7—N2—C8	−3.5 (3)
N1—C5—C6—C8	2.1 (3)	N1—C7—N2—C9	178.92 (19)
C1—C6—C8—O1	−6.6 (3)	O1—C8—N2—C7	−172.35 (19)
C5—C6—C8—O1	172.9 (2)	C6—C8—N2—C7	6.3 (3)
C1—C6—C8—N2	174.76 (17)	O1—C8—N2—C9	5.2 (3)
C5—C6—C8—N2	−5.7 (3)	C6—C8—N2—C9	−176.13 (16)
C14—C9—C10—C11	−0.8 (3)	C10—C9—N2—C7	44.8 (3)
N2—C9—C10—C11	−179.52 (18)	C14—C9—N2—C7	−133.95 (19)
C9—C10—C11—C12	−1.4 (3)	C10—C9—N2—C8	−132.82 (19)
C10—C11—C12—C13	2.3 (3)	C14—C9—N2—C8	48.5 (3)

### Hydrogen-bond geometry (Å, °)

**Table:**

*D*—H···*A*	*D*—H	H···*A*	*D*···*A*	*D*—H···*A*
C7—H7···N1^i^	0.93	2.47	3.281 (2)	145
C13—H13···O1^ii^	0.93	2.37	3.145 (2)	140

Symmetry codes: (i) −*x*+1, −*y*+2, −*z*+1; (ii) −*x*+3/2, *y*+1/2, −*z*+1/2.
